# Central venous Access device SeCurement And Dressing Effectiveness for peripherally inserted central catheters in adult acute hospital patients (CASCADE): a pilot randomised controlled trial

**DOI:** 10.1186/s13063-017-2207-x

**Published:** 2017-10-04

**Authors:** Raymond J. Chan, Sarah Northfield, Emily Larsen, Gabor Mihala, Amanda Ullman, Peter Hancock, Nicole Marsh, Nicole Gavin, David Wyld, Anthony Allworth, Emily Russell, Md Abu Choudhury, Julie Flynn, Claire M. Rickard

**Affiliations:** 1 Royal Brisbane and Women’s Hospital, Metro North Hospital and Health Service, Butterfield Street, Herston, Brisbane, Queensland 4029 Australia; 2 School of Nursing and Institute of Health and Biomedical Innovation, Queensland University of Technology, Kelvin Grove, Brisbane, Queensland 4059 Australia; 3 Alliance for Vascular Access Teaching and Research (AVATAR) Group, NHMRC Centre of Research Excellence in Nursing, Menzies Health Institute Queensland, Griffith University, Nathan, Brisbane, Queensland 4111 Australia; 4 School of Medicine, University of Queensland, Herston, Brisbane, Queensland 4029 Australia

**Keywords:** Peripherally inserted central catheter (PICC), Dressing and securement methods, Complications, Randomised controlled trials, Vascular access devices, Catheterization, Central venous

## Abstract

**Background:**

Peripherally inserted central catheters (PICCs) are commonly used for delivering intravenous therapy. PICC failure is unacceptably high (up to 40%) due to mechanical, infectious and thrombotic complications. Poor securement potentiates all complication types. This randomised controlled trial (RCT) aimed to examine the feasibility of a large RCT of four dressing and securement methods to prevent PICC failure.

**Methods:**

This single-centre pilot RCT included 124 admitted medical/surgical/cancer patients aged ≥ 16 years with a PICC. Interventions were: (i) standard polyurethane dressing and sutureless securement device (SPU + SSD, control); (ii) polyurethane with absorbent lattice pad dressing (PAL + Tape); (iii) combination securement-dressing (CSD); and (iv) tissue adhesive (TA + SPU). All groups except TA + SPU had a chlorhexidine-gluconate (CHG) impregnated disc. Feasibility outcomes were recruitment and safety/acceptability of the interventions. The primary outcome was PICC failure, a composite of PICC removal for local infection, catheter-associated bloodstream infection, dislodgement, occlusion, and/or catheter fracture. Secondary outcomes included individual complications, dressing failure and dwell time, PICC dwell time, skin complications/phlebitis indicators, product costs, and patient and staff satisfaction. Qualitative feedback was also collected.

**Results:**

PICC failure incidence was: PAL + CHG + Tape (1/5; 20%; 17.4/1000 days), SPU + SSD + CHG (control) (4/39; 10%; 9.0/1000 days), TA + SPU (3/35; 9%; 9.6/1000 days), and CSD + CHG (3/42; 7%; 9.4/1000 days). Recruitment to PAL + CHG + Tape was ceased after five participants due to concerns of PICC dislodgement when removing the dressing. CSD + CHG, TA + SPU (TA applied only at PICC insertion time), and control treatments were acceptable to patients and health professionals.

**Conclusion:**

A large RCT of CSD + CHG and TA + SPU (but not PAL + CHG + Tape) versus standard care is feasible.

**Trial registration:**

Australian and New Zealand Clinical Trials Registry, ACTRN12616000027415. Registered on 15 January 2016.

## Background

Peripherally inserted central catheters (PICCs) are commonly used medical devices for life-saving treatment in the hospital [1]. Generally inserted into large deep veins in the upper periphery (basilic, brachial, cephalic, axillary), they are threaded to the superior vena cava for blood sampling, medications, fluids, nutrition, and blood transfusion [2]. PICCs suit prolonged and/or frequent therapy, vesicant chemotherapy, and irritant infusions, especially in patients with limited veins [3].

Despite their critical role, PICC failure rates are unacceptably high (up to 40%), interrupting therapy [4–7] due to mechanical, infectious, and thrombotic complications. Catheter migration (movement of the device from its central placement) is a key clinical concern that can potentially result in infiltration and extravasation. Thrombosis is also a major issue, causing pain and risk of embolus [8]. Such scenarios can lead to infiltration or accidental dislodgement of the catheter. Catheter-associated bloodstream infection (CABSI) is a serious health-care-associated infection that can potentially result in septic embolism, infective endocarditis, osteomyelitis, septic arthritis, and death [9–11]. Furthermore, PICC failures cause negative patient experiences, including painful repeated needle-sticks, and increases in hospital length of stay, equipment costs, and workloads [12].

Dressing and securement methods are integral in preventing PICC failure and complications, yet current dressings are often inadequate [13]. Effective methods should prevent PICCs from dislodging or migrating, and provide a protective barrier from microbial colonisation and infection. Good dressing and securement methods also minimise micro-motion, risk of vein thrombosis, and PICC fracture (or other damage to the PICC). There are a number of dressing and securement devices available for PICCs [13, 14], and there are variable applications within clinical practice [13]. A recent Cochrane review [14] of 22 randomised controlled trials (RCTs) (*n* = 7436 patients with central venous access devices (CVADs)) found medication-impregnated dressing products to be more effective than non-medicated dressings to prevent catheter-related bloodstream infections (CRBSI). However, only five of the included trials studied PICCs, most were conducted in intensive care units (ICUs), and many dressing products had no evidence or low-quality evidence on effectiveness, thus high uncertainty remains. The authors concluded that high-quality research is required to assess the effectiveness of novel products [14]. This study aimed to provide feasibility data for a fully powered RCT comparing standard care versus three innovative dressing and securement methods for PICCs in adult patients receiving acute care.

## Methods

### Study location

This trial was conducted at the Royal Brisbane and Women’s Hospital (RBWH), the largest quaternary referral hospital in Queensland, Australia.

### Patients and procedures

Patients were recruited from March 2014 to March 2015. Inclusion criteria were ≥ 16 years of age; planned for PICC insertion; expected inpatient stay >24 hours; and informed consent. Exclusions were current bloodstream infection (BSI) (within 48 hours) at time of PICC insertion; non-English speaking without an interpreter; diseased, burned or scarred skin; skin tear or “paper” skin at the insertion site; allergy to any study product; enrolled in a competing CVAD study or previously enrolled in this study. ICU patients were not studied as PICCs are rarely used in Australian ICUs. We aimed to recruit 30 participants per arm to provide estimates of relative treatment effects and assess protocol feasibility [15, 16]. Initially, only patients with cancer were targeted and only pre-PICC insertion consent requested but recruitment was slow due to: (1) the size of the potential recruitment pool; (2) urgent insertions without time to request consent for the study; (3) unpredictable changes in scheduling of PICC insertions; and (4) patients having PICCs inserted in two hospital departments at the same time, meaning the research nurse was not always present. After 2 weeks, we extended the trial to medical and surgical departments, and extended the recruitment window to 24 hours post PICC insertion if no PICC complications had occurred (in these patients, the original dressing was carefully removed and replaced with the randomised dressing). The ethics committee approved this amendment.

#### Recruitment, randomization, allocation concealment, and blinding

Research Nurses (ReNs) screened patients daily using a screening log, gained informed consent, and performed randomization after consent. A centralized web-based service maintained allocation concealment and randomised participants in a 1:1:1:1 ratio between the four groups, with block sizes randomly varied as blocks of 4 or 8 (https://www151.griffith.edu.au/ctcc). Patients, clinical staff or ReNs were not blinded due to the nature of the intervention. However, the infectious diseases physician classifying CABSI and local infection outcomes was blinded. Other outcomes (e.g. dislodgement) were assigned by clinical, not research staff or investigators, using routine clinical practices and documentation, which minimised the risk of bias influencing outcomes. A Study Manager trained and supervised the ReNs, and undertook quality checks on data collected. There was a 54-week recruitment period, with a maximum 4-week follow-up period from insertion (or earlier if the device removed or the patient was discharged).

#### Ethical considerations

The RBWH Human Research Ethics Committee (HREC) (HREC/13QRBW/454) and the Griffith University HREC (NRS/10/14/HREC) approved the study on 10 February and 20 February 2014, respectively. The trial was registered with the Australian and New Zealand Clinical Trials Registry: ACTRN12616000027415. All products were registered with the Australian Therapeutic Goods Administration. Adverse events (i.e. skin complications: any rash, blister, itchiness, skin tear, or bruising) were recorded. Serious adverse events (e.g. BSI, ICU admissions, or death) were reported to the HREC. Written informed consent was obtained from participants/substitute decision-makers or the next of kin for those younger than 18 years of age. Each participant, substitute decision-maker or next of kin was provided with ample time to read and consider the participant information sheet before signing the consent form. Informed consent and signature was witnessed by an independent party and the ReN.

### Study groups

**Fig. 1:**
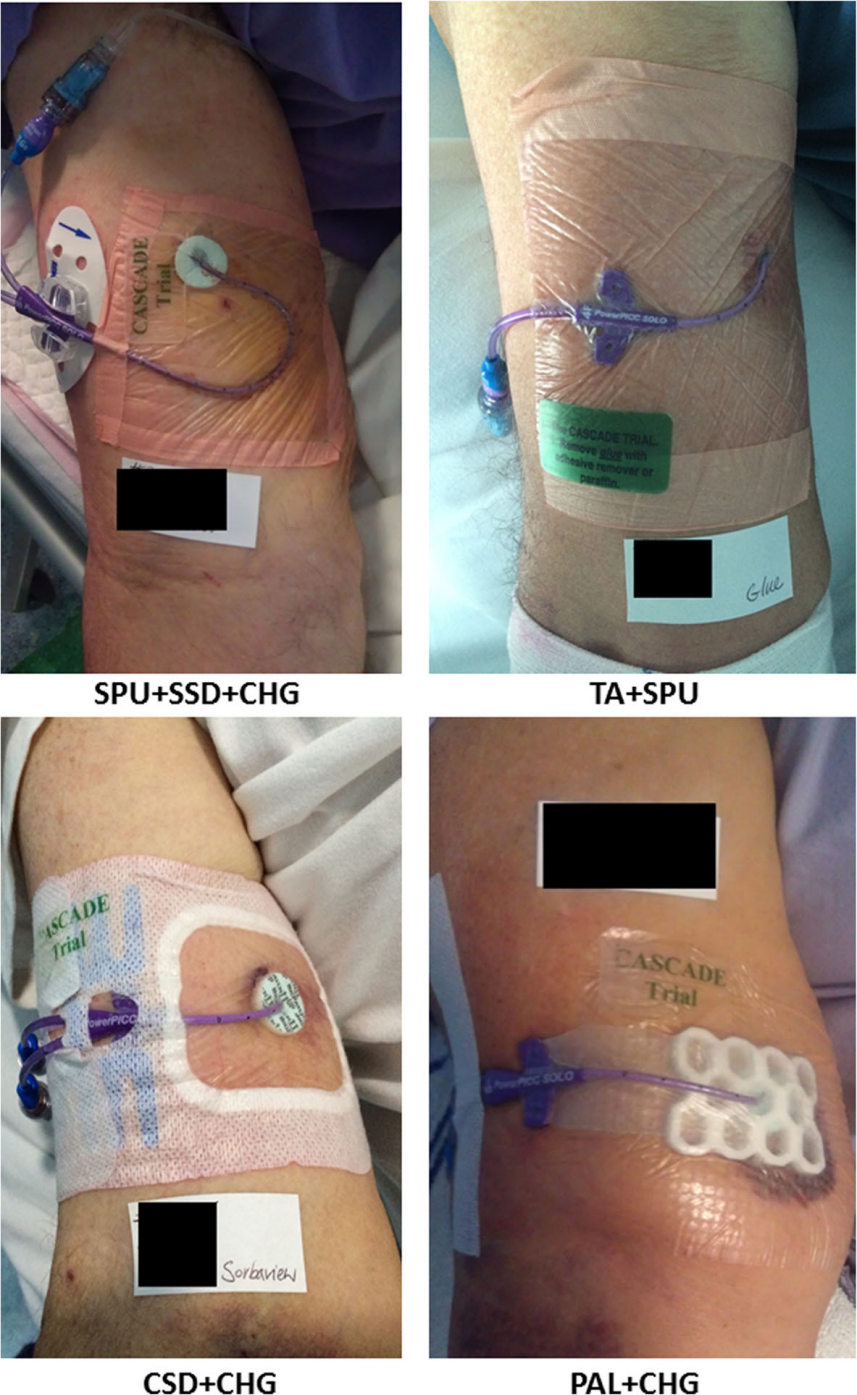
Dressing and securement methods for each study group. SPU + SSD, standard polyurethane dressing plus a sutureless securement device plus chlorhexidine-gluconate impregnated discs; PAL + CHG + Tape, polyurethane with absorbent lattice pad adhesive plus non-woven tape plus chlorhexidine-gluconate impregnated discs; CSD + CHG, combination securement dressing plus chlorhexidine-gluconate impregnated discs; TA + SPU, tissue adhesive plus standard polyurethane dressing

Group 1 (control): received a standard polyurethane dressing (SPU) [17–21] (IV3000™ - Standard 10 cm x 14 cm [Product ID: 18477493]; Smith and Nephew, Hull, UK) and a sutureless securement device (SSD) (StatLock™ PICC Plus Stabilization Device [Product ID: VPPDFP]; Bard Medical, Covington, GA, USA) placed outside of the dressing area as per hospital policy [21–24]. Group 2 received a polyurethane with absorbent lattice pad (PAL) dressing (OPSITE™ Post-Op Visible® 9 cm x 10 cm  [Product ID: 6600842] [25]; Smith and Nephew, Hull, UK) and adhesive, non-woven tape (Fixomull® 10 cm x 10 cm [Product ID: 02037-00] ; BSN Medical, Hamburg, Germany). The PAL dressing “sandwiches” the PICC with two strips of polyurethane (one on top and one underneath the PICC) to provide additional securement. Group 3 received a combination securement dressing (CSD) (Sorbaview SHIELD® 9.5 cm x 14 cm [Product ID: SV353UDT-6] [26]; Centurion, Williamston, MI, USA), which has increased securement under and over the PICC and an absorbent layer. Group 4 received tissue adhesive (TA) (Histoacryl Blue® [Product ID: TS1050044FP] [27, 28]; B Braun Surgical, Rubi, Spain), two to three drops at the insertion site and under the PICC wings and allowed 30 s to dry, plus SPU as above. With the exception of the TA group, all groups used chlorhexidine-gluconate impregnated discs (CHG) at the PICC insertion site (Biopatch® [Product ID: 4151]; Johnson and Johnson, NJ, USA). The study groups were referred to as SPU + SSD + CHG, PAL + CHG + Tape, CSD + CHG, and TA + SPU, respectively. Figure 1 shows all dressing and securement methods.

#### Insertion and care of the PICC and securements

PICCs were inserted by clinical nurses, radiographers and radiologists following skin decontamination with swab sticks impregnated with 2% chlorhexidine gluconate and 70% isopropyl alcohol. If patients had a known skin allergy to this product, skin decontamination was performed using Betadine solution (containing 10% povidone-iodine). The ultrasound/fluoroscopy-guided insertion and chest x-ray tip confirmation techniques were used. Personnel performing the insertion or ReNs applied the allocated products at insertion or within 24 hours. SmartSite® needleless connectors were applied as standard to all PICCs. All clinical nurses and ReNs at the study site must achieve clinical competencies in PICC management to perform dressing changes, as per hospital policy. All clinical nurses, radiographers, radiologists, and ReNs involved in the study were equally trained in dressing application and removal in all intervention groups. The ReNs provided step-by-step written and verbal instructions to all healthcare professionals involved in the study on applying and removing dressings in all intervention groups, and were available to provide assistance in changing dressings during business hours. Study products were changed by clinical nurses with or without the study ReN weekly, and as needed, to remain clean, dry and intact [23]. TA was removed with Remove© wipes (Smith and Nephew) then reapplied at each dressing change. In this pragmatic trial, clinical nurses added strips of non-sterile paper tape to infusion tubing and additional securement products at their discretion, and these were recorded by ReNs.

For suspected CABSI, clinical (not research) staff ordered peripheral blood cultures with or without PICC-drawn qualitative (not quantitative) blood cultures, as per usual practice [29]. PICC blood culture, entry site and tip cultures were taken at clinician discretion. Blood cultures were not taken routinely as they provide no added diagnostic value to those taken on clinical suspicion of infection [30, 31].

#### Data collection

Data were directly entered into Research Electronic Data CAPture (REDCap; Vanderbilt University, hosted by Griffith University) by ReNs using a tablet computer. Baseline data were collected on demographic and clinical characteristics (age, gender, diagnosis, immunosuppression, infection status, co-morbidities, weight, skin type/integrity, hand dominance (right/left)); and on device characteristics (location, side (right/left), number of lumens, inserter, number of attempts, skin preparation, and infusates). ReNs visited active patients daily to assess protocol compliance, and to document endpoints and adverse events. PICC removal data were collected on the reason for PICC removal, site complications, dwell time, length of stay, mortality, level of consciousness, mobility, and cognitive state. Data on product residue, rash, blister, itchiness, or tearing of skin on dressing removal were recorded.

We reviewed cases of CABSI for CRBSI using the differential time to positivity, or matched tip/blood culture criteria [32]. All microbiology analysis was by blinded scientists, and endpoints were adjudicated by a blinded infectious diseases physician. Product residue, rash, blister, itchiness, or tearing of skin on dressing removal were recorded. A purposive sample of 20 clinical staff (who applied or removed study products) participated in brief semi-structured interviews about the strengths and weaknesses of any study products that they had used during the trial.

#### Outcomes

Feasibility outcomes were recruitment, safety, and acceptability of the interventions. The primary endpoint was PICC failure, a composite of reasons for premature PICC removal (i.e. PICC could no longer be used for required therapy). This was recorded dichotomously per patient (yes/no) and expressed as incidence rates per 1000 PICC days, to account for the varying PICC dwell durations and thus exposure to the chance of developing a failure outcome. PICC failure is a common measure in vascular access research [5] and includes: (1) CABSI (laboratory-confirmed bloodstream infection not related to another site) [33]; (2) local infection (positive tip/skin colonisation around the exit site, purulent discharge, or redness extending 1 cm beyond the site that prompts the clinician to order removal and commence antimicrobial therapy); (3) dislodgement: *total* – the PICC lumen tip completely leaves the vein; *partial* – the PICC tip is no longer in the superior vena cava (diagnosed by chest x-ray, leakage from the site on injection, or clinician diagnosis); post-insertion change in PICC length (catheter marking) at the hub; (4) occlusion: ≥1 lumen unable to be flushed/aspirated, as diagnosed by clinician; [34] and/or (5) PICC fracture: visible fracture, leak, split, or other damage to the PICC material as diagnosed by the treating clinician.

Secondary outcomes were each type of PICC failure; dressing/securement failure (early replacement before 7 days as loose, soiled, or missing); PICC dwell time and first dressing/securement dwell time: hours from insertion/application until removal; skin complications and phlebitis indicators; purchase costs of study products for one application/per patient use of each dressing/securement (does not include labour costs). Patient satisfaction data were collected upon completion of the study (0 = totally dissatisfied; 10 = totally satisfied). Nurses who removed the study dressing at the completion of the study were asked to rate the degree of difficulty in completing this task (0 = extremely difficult; 10 = extremely easy).

### Statistical analysis

Feasibility outcomes and qualitative data were reported descriptively. All randomised patients were analyzed using intention-to-treat analysis. Patients were the unit of measurement (one PICC per patient). Prior to analysis (Stata 14.1, Stata-Corp), outlying figures, missing data, and implausible data were cleaned, and a random 5% source data rechecked. Missing values were not imputed. Incidence rates per 1000 PICC days and rate ratios were calculated to test differences between groups, with comparisons over time using Kaplan-Meier survival curves and log-rank tests. Secondary endpoints were reported using frequencies, proportions or mean (SD), or median (interquartile range). Cost data per dressing change were reported descriptively. Cox regression was used to test the effect of covariates (variables <0.2 on univariable analysis were included in the multivariable model), and the effect of study group on PICC failure. Continuous variables (e.g. age) were centred over means and correlations between covariates were checked. The categorical covariates were re-grouped for the Cox multivariable analysis, by merging small (n < 20) categories into neighbouring categories with similar effects, or similarly merging categories to explore where the significant cutoff point was. The ten-events-per-variable rule was considered and the final model derived by manual backward deletion of covariates at *p* ≥ 0.05 (keeping study group in the model). The proportional-hazards assumption was checked using the Schoenfeld residuals on the final multivariable model. The final multivariable model breached our own self-imposed ten-events-per-variable rule, which was done with the intention to inform future studies (otherwise potentially interesting associations would not show up), and was deemed acceptable for a pilot study [35]. No adjustment was made for multiple comparisons. *P* < 0.05 was considered significant.

## Results

### Feasibility outcomes and participant characteristics

**Fig. 2:**
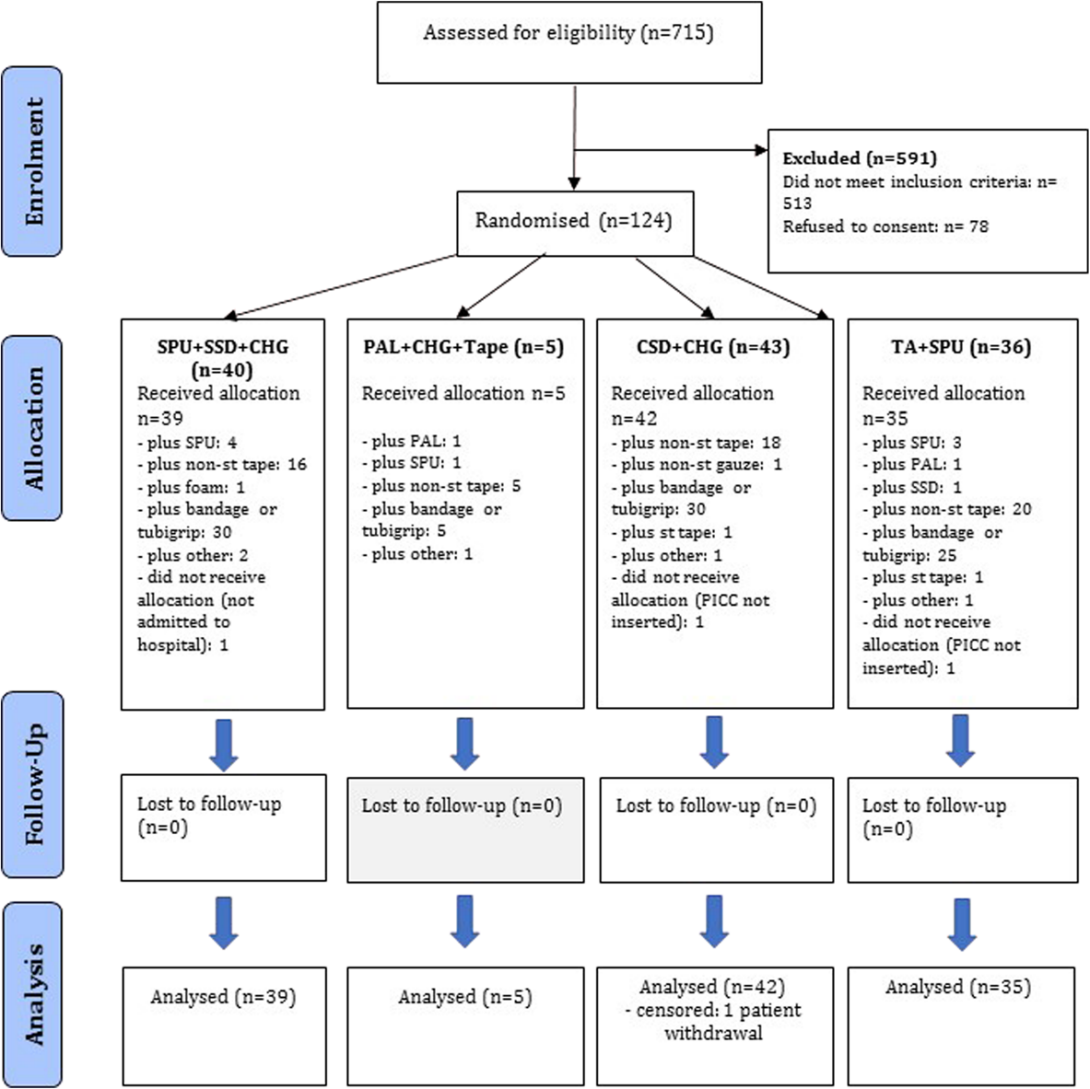
Consolidated Standards of Reporting Trials (CONSORT) flow chart. SPU + SSD, standard polyurethane dressing plus a sutureless securement device plus chlorhexidine-gluconate impregnated discs; PAL + CHG + Tape, polyurethane with absorbent lattice pad adhesive plus non-woven tape plus chlorhexidine-gluconate impregnated discs; CSD + CHG, combination securement dressing plus chlorhexidine-gluconate impregnated discs; TA + SPU, tissue adhesive plus standard polyurethane dressing

Of 715 patients screened for eligibility, 124 were recruited and randomised; the outcomes of 121 patients (98%) were analyzed with the exception of 3 patients unable to have a PICC inserted or who had no further study involvement (Fig. 2). One patient (CSD + CHG group) withdrew from the study but allowed censored outcome data to be included in the analysis. At baseline, the groups were generally similar in demographic and clinical factors (Table 1). Participants in all groups, reported mean satisfaction scores >8 out of 10 (Table 2). Clinicians reported PAL + CHG + Tape as high risk for PICC dislodgement at both insertion and removal in each of the five patients studied, due to the seamless “sandwich” component of the dressing, with one PICC dislodged. Due to clinical staff concern, recruitment to this group ceased. The TA + SPU technique was found problematic after repeated applications (at each dressing replacement) with build-up occurring on the PICC; this was not observed after the initial application. There was no negative feedback on the CSD. Many patients in all groups received additional PICC reinforcement at the discretion of clinical staff; this was permitted as this was a pragmatic trial (Fig. 2). A total of 1132 catheter-days were studied, with 6.9 days median PICC dwell per patient.

**Table 1:** Participant, PICC insertion and treatment characteristics at randomisation*

	Group 1 SPU + SSD+ CHG	Group 2 PAL+ CHG + Tape	Group 3 CSD+ CHG	Group 4 TA + SPU
At recruitment (*N* = 124)	(*n* = 40)	(*n* = 5)	(*n* = 43)	(*n* = 36)
Age (years)^a^	56 (16)	63 (20)	57 (15)	54 (18)
Sex (male)	25 (62)	2 (40)	27 (63)	25 (69)
Overweight/obese (*n* = 102)	20 (62)	2 (50)	21 (58)	16 (53)
Skin integrity:				
- good	13 (32)	1 (20)	13 (30)	14 (39)
- fair	21 (52)	3 (60)	26 (60)	18 (50)
- poor	6 (15)	1 (20)	4 (9)	4 (11)
Skin type (white)	32 (80)	3 (60)	30 (70)	26 (72)
Comorbidities	31 (78)	4 (80)	32 (74)	21 (58)
- none	3 (8)	1 (20)	4 (9)	5 (14)
- one	6 (15)	0 (0)	7 (16)	10 (28)
- two	6 (15)	1 (20)	7 (16)	5 (14)
- three	6 (15)	1 (20)	6 (14)	2 (6)
- four or more	19 (48)	2 (40)	19 (44)	14 (39)
Diagnosis:				
- medical	10 (25)	1 (20)	14 (33)	10 (28)
- surgical emergency	9 (22)	2 (40)	11 (26)	8 (22)
- surgical elective	8 (20)	0 (0)	6 (14)	8 (22)
- haematology	4 (10)	1 (20)	4 (9)	7 (19)
- oncology	5 (12)	1 (20)	3 (7)	3 (8)
- surgical oncology	4 (10)	0 (0)	5 (12)	0 (0)
Leucocytes low^b^	1 (2)	0 (0)	0 (0)	0 (0)
Infection (any)	23 (58)	4 (80)	24 (56)	20 (56)
Antibiotic therapy:	31 (78)	5 (100)	30 (70)	28 (78)
- intravenous	31 (78)	3 (60)	29 (67)	27 (75)
- oral	1 (2)	3 (60)	8 (19)	4 (11)
At PICC insertion (*N* = 121)	(*n* = 39)	(*n* = 5)	(*n* = 42)	(*n* = 35)
PICC type (*n* = 120):				
- basilic	35 (92)	5 (100)	34 (81)	33 (94)
- brachial	2 (5)	0 (0)	5 (12)	1 (3)
- cephalic	1 (3)	0 (0)	3 (7)	1 (3)
Subsequent insertion	11 (28)	0 (0)	12 (29)	8 (23)
Inserted by:				
- nurse	36 (92)	5 (100)	35 (83)	31 (89)
- radiographer	3 (8)	0 (0)	7 (17)	3 (9)
- doctor	0 (0)	0 (0)	0 (0)	1 (3)
Insertion method:				
- ultrasound	27 (69)	5 (100)	32 (76)	22 (63)
- ultrasound, fluoroscopy	12 (31)	0 (0)	10 (24)	13 (37)
Multiple insertion attempts	5 (13)	1 (20)	3 (7)	5 (14)
Hair, before insertion:	4 (10)	0 (0)	10 (24)	8 (23)
- if yes, clipped^c^	2	-	1	2
Inserted on dominant side	20 (51)	3 (60)	22 (52)	19 (54)
Number of lumens (two)^d^	27 (69)	1 (20)	31 (74)	23 (66)
Treatment (*N* = 121)	(*n* = 39)	(*n* = 5)	(*n* = 42)	(*n* = 35)
IV fluids/medications^e^:				
- fluid continuous	29 (74)	4 (80)	31 (74)	31 (89)
- normal saline flush	11 (28)	2 (40)	5 (12)	3 (9)
- chemotherapy	3 (8)	1 (20)	3 (7)	6 (17)
- PN (non-lipid)	6 (15)	0 (0)	8 (19)	5 (14)
- lipids	7 (18)	0 (0)	6 (14)	4 (11)
- blood products	11 (28)	2 (40)	7 (17)	8 (23)
- antibiotics	31 (79)	2 (40)	28 (67)	29 (83)
- heparin, continuous	5 (13)	0 (0)	2 (5)	5 (14)
Patient confused/agitated/drowsy (*n* = 119)^f^	1 (3)	0 (0)	6 (15)	4 (12)
Ventilated/intubated (*n* = 119)^f^	1 (3)	0 (0)	1 (2)	0 (0)
Independently mobile (*n* = 119)^f^	22 (56)	3 (60)	25 (61)	21 (62)
Other VAD in situ^f^	2 (5)	0 (0)	9 (21)	9 (26)

*Data presented on 124 randomised patients and 121 inserted peripherally inserted central catheters (PICCs); number (percentage) shown unless otherwise noted. Frequencies may not add up to group size (and frequencies to 100%) due to missing data

*SPU* simple polyurethane dressing, *SSD* sutureless securement device, *CHG* chlorhexidine gluconate-impregnated discs, *PAL* polyurethane with absorbent lattice pad dressing, *CSD* combination securement dressing, *TA* tissue adhesive, *VAD* vascular access device, *PN* parenteral nutrition

^a^Mean (standard deviation) shown

^b^Absolute value <1000 per microlitre

^c^Frequencies shown only

^d^Other categories omitted

^e^Received at any time during the study period

^f^At device removal

**Table 2:** Study outcomes (*n* = 121)

	Group 1 SPU + SSD + CHG (*n* = 39)	Group 2 PAL + CHG + Tape (*n* = 5)	Group 3 CSD + CHG (*n* = 42)	Group 4 TA + SPU (*n* = 35)
PICC failure (per patient)	4 (10%)	1 (20%)	3 (7%)	3 (9%)^a^
Incidence rate (95% CI)^b^	9.03 (3.39–24.1)	17.4 (2.45–123)	9.43 (3.04–29.2)	9.57 (3.09–29.7)
Rate ratio (95% CI)	referent	1.92 (0.04–19.4)	1.04 (0.15–6.17)	1.06 (0.16–6.27)
Log-rank test (*p* value)	referent	0.629	0.905	0.939
Catheter-days	443	58	318	313
PICC dwell time (days)^c^	8.94 (3.13, 20.9)	9.99 (7.23, 16.1)	5.56 (4.11, 9.97)	7.11 (4.00, 14.8)
Secondary outcomes:				
- CABSI	0 (0%)	0 (0%)	2 (5%)	1 (3%)
- dislodgement	4 (10%)	1 (20%)	0 (0%)	2 (6%)
- occlusion	0 (0%)	0 (0%)	1 (2%)	0 (0%)
- fracture	0 (0%)	1 (20%)	0 (0%)	1 (3%)
- 1^st^ secdev life <7d (*n* = 116)	26 (72%)	4 (80%)	25 (61%)	25 (74%)
- patient satisfaction^d,e^	8.79 (1.67)	9.25 (1.50)	9.17 (1.48)	8.17 (2.02)
- difficulty of removal^d,f^	8.44 (2.14)	5.00 (4.36)	7.97 (2.38)	6.04 (2.74)
Skin complication^g^	12 (30%)	1 (20%)	9(21%)	13 (36%)
Phlebitis indicators (*n* = 119):^h^				
- pain ≥2/10	2 (5%)	0 (0%)	4 (10%)	5 (14%)
- tenderness ≥2/10	5 (13%)	0 (0%)	8 (20%)	10 (28%)
- erythema (any)	7 (18%)	0 (0%)	8 (20%)	11 (31%)
- swelling (any)	1 (3%)	1 (20%)	3 (7%)	9 (26%)
- purulent discharge	0 (0%)	1 (20%)	1 (2%)	2 (6%)
- any	11 (29%)	2 (40%)	17 (41%)	18 (51%)
Serious adverse events:				
- death	0 (0%)	0 (0%)	1 (2%)	2 (6%)
- positive blood culture	0 (0%)	0 (0%)	2 (5%)	2 (6%)
- other	2 (5%)	0 (0%)	1 (2%)	1 (3%)
Number of dressing changes:	72	8	52	60
- Incidence rate^b^	163	139	163	191
Dressing/secdev life:				
- days to first change^c^	1.71 (0.66, 3.38)	0.94 (0.41, 2.60)	1.83 (0.53, 5.45)	1.49 (0.56, 3.44)
- days^d^	3.68 (1.77)	5.21 (2.86)	3.53 (1.98)	3.41 (1.52)
Reason for change (*n* = 189)^i^:				
- routine	45 (62%)	3 (38%)	27 (53%)	19 (33%)
- dressing lifting	19 (26%)	4 (50%)	11 (22%)	27 (47%)
- sweating	4 (6%)	0 (0%)	0 (0%)	2 (3%)
- leakage	2 (3%)	2 (25%)	1 (2%)	0 (0%)
- bleeding	12 (17%)	5 (62%)	11 (21%)	10 (17%)
- unknown	0 (0%)	0 (0%)	0 (0%)	1 (2%)
- other	18 (25%)	2 (25%)	20 (39%)	28 (48%)

Number (percentage) shown unless otherwise noted

*SPU* simple polyurethane dressing, *SSD* sutureless securement device, *CHG* chlorhexidine gluconate-impregnated discs, *PAL* polyurethane dressing with absorbent lattice pad, *CSD* combination securement dressing, *TA* tissue adhesive, *PICC* peripherally inserted central catheter; *CABSI* catheter associated bloodstream infection, *secdev* securing device, *VAD* vascular access device

^a^One patient had two forms of failure (dislodgement and breakage) but was only counted once

^b^Incidence rate per 1000 catheter-days

^c^Median and interquartile range shown as 25^th^ and 75^th^ percentiles

^d^Mean and standard deviation

^e^Patient self-report, 0 = completely dissatisfied, …, 10 = completely satisfied;

^f^Nurse rating of difficulty when removing the product: 0 = very difficult, …, 10 = very easy; ^g^Any of rash, blister, itchiness, skin tear, or bruising at device removal; values may not add up to total due to rounding, percentages were calculated with the number of non-missing values in the denominator

^h^Observed at any time during study

^i^Denominator: number of dressing changes in that group; frequencies may not add up to group size (and frequencies to 100%) due to missing data

### Primary outcomes

**Fig. 3:**
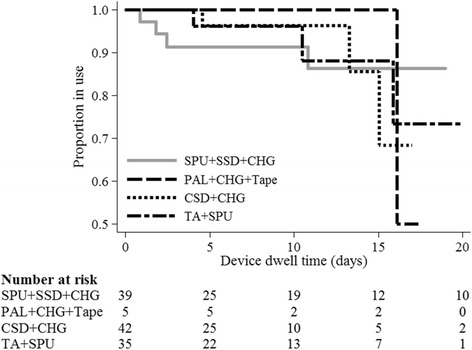
Kaplan-Meier survival estimates from peripherally inserted central catheter (PICC) failure, by study group; log-rank test, *p* = 0.98; *n* = 121. SPU + SSD, standard polyurethane dressing plus a sutureless securement device plus chlorhexidine-gluconate impregnated discs; PAL + CHG + Tape, polyurethane with absorbent lattice pad adhesive plus non-woven tape plus chlorhexidine-gluconate impregnated discs; CSD + CHG, combination securement dressing plus chlorhexidine-gluconate impregnated discs; TA + SPU, tissue adhesive plus standard polyurethane dressing

Catheter failure rate was highest in PAL + CHG + Tape (1/5, 20%), and lowest in CSD + CHG (3/42, 7%), with SPU + SSD + CHG (control) and TA + SPU having failure rates of 10% (4/39) and 9% (3/35), respectively (Table 2). Incident rates per 1000 catheter-days were 17, 9, 9, and 10, respectively (log-rank test, *p* = 0.980) (Table 2 and Fig. 3). The hypothesis of no difference in failure over time was not rejected. The adjusted hazard ratio (HR) of PICC failure compared to control was one third lower in CSD + CHG (HR = 0.64, 95% CI = 0.14–2.92), similar in TA + SPU (HR = 1.07, 95% CI = 0.24–4.83) and increased fourfold in PAL + CHG + Tape (HR = 4.17, 95% CI = 0.36–48.4) (Table 3). PICC failure was approximately 90% less likely in women than in men (HR = 0.10, 95% CI = 0.01–0.87, *p* = 0.037), but increased fourfold in patients with three or more comorbidities, compared to those with one or no comorbidities (HR = 4.62, 95% CI = 1.04–20.4, *p* < 0.005).

**Table 3:** Cox regression of PICC failure (*n* = 121)

	Univariable HR (95% CI)	Multivariable HR (95% CI)
Intervention (referent (ref) SPU + SSD + CHG):		
- PAL + CHG + Tape	1.60 (0.18–14.4)	4.17 (0.36–48.4)
- CSD + CHG	1.06 (0.23–4.83)	0.64 (0.14–2.92)
- TA + SPU	1.04 (0.23–4.67)	1.07 (0.24–4.83)
Age (one year older)	1.02 (0.97–1.06)	–
Female sex (ref. male)	0.15 (0.02–1.15)*	0.10 (0.01–0.87)**
Overweight/obese (ref. other)	1.70 (0.47–6.20)	–
Comorbidities (ref. up to 3)	3.37 (0.89–12.8)*	4.62 (1.04–20.4)**
Skin integrity fair/poor (ref. good)	2.00 (0.43–9.28)	–
Skin type brown (ref. white)	0.67 (0.14–3.12)	–
Diagnosis		–
- oncology (ref. surgical)	1.50 (0.43–5.20)	–
- medical (ref. surgical)	0.36 (0.04–3.09)	–
Gastrointestinal surgery (ref. other type)	0.90 (0.16–4.94)	–
Drain (ref. none)	2.10 (0.61–7.19)	–
Wound (ref. none)	0.91 (0.24–3.43)	–
Infection (ref. none)	1.14 (0.33–3.91)	–
IV therapy (ref. none)	*a*	–
Antibiotic therapy (ref. none)	0.75 (0.20–2.84)	–
U/S guided insertion with fluoroscopy (ref. U/S guided)	2.34 (0.71–7.70)*	*b*
Insertion on dominant side (ref. false)	1.07 (0.32–3.51)	–
Lumens (one more)	*a*	–
Subsequent device (ref. false)	2.68 (0.80–8.92)*	*b*
Device length (1 cm longer)	1.00 (0.91–1.11)	–

Baseline observations were used; *a* = cannot be calculated; *b* = removed during variable selection at *p* ≥ 0.05; hyphen = not entered into the multivariate model

*PICC* peripherally inserted central catheter, *HR* hazard ratio, *CI* confidence interval, *SPU* simple polyurethane dressing, *SSD* sutureless securement device, *CHG* chlorhexidine gluconate-impregnated discs, *PAL* polyurethane with absorbent lattice pad dressing, *CSD* combination securement dressing, *TA* tissue adhesive, *U/S* ultrasound

*p value <0.20; **p value <0.05

### Secondary outcomes

Secondary outcomes are reported in Table 2. PICC failure was most commonly due to dislodgement, affecting 6% of all patients. Three participants developed CABSI (CSD + CHG, *n* = 2; TA + SPU, *n* = 1). Of these, one (CSD + CHG group) was confirmed as CRBSI by differential time to positivity of peripheral and PICC-drawn blood cultures [32]. There were no local infections. Median days to first dressing change was shortest in PAL + CHG + Tape (0.9 days, 95% CI = 0.41–2.60), and longest in CSD + CHG (1.8 days, 95% CI = 0.53–5.45). Average dressing dwell was shortest in the TA + SPU and CSD + CHG groups (3.4 days and 3.5 days, respectively). The purchase costs (AUD 2015) of dressing and securements per one application/per patient for all applications were: AU$18.52/AU$34.66 (SPU + SSD + CHG); AU$18.36/AU$33.05 (PAL + CHG + Tape), AU$16.33/AU$20.61 (CSD + CHG) and AU$18.82/AU$32.80 (TA + SPU).

Skin complications as a composite outcome of any rash, blister, itchiness, skin tear, or bruising at device removal ranged from 20% to 36% across study groups (Table 2). TA + SPU had the most participants with phlebitis indicators including pain (*n* = 5, 14%), tenderness (*n* = 10, 28%), erythema (*n* = 11, 31%), swelling (*n* = 9, 26%), and purulent discharge (*n* = 2, 6%) (Table 2). Nursing staff reported that SPU + SSD + CHG products were the easiest to remove (mean = 8.44 out of 10) and PAL + CHG + Tape were the most difficult (mean = 5.00 out of 10).

## Discussion

Feasibility outcomes were generally positive with 10 patients randomised on average each month, if patients from all specialties (medical, surgical, and cancer care) were included. Attrition was low at 1/121 (0.8%) and there were no missing data for the primary endpoint. The standard care (control), CSD + CHG group and the TA + SPU group (with use at insertion only) appear feasible and acceptable for future trials. The CSD + CHG group, with the “two-in-one” CSD, had the least failure (7%); a larger trial would be needed to detect statistical differences. Purchase costs per patient in this group were 40% less than for controls and future trials should now assess cost-effectiveness. Both clinician satisfaction scores and qualitative feedback indicated CSD was the easiest product to apply and remove. While the PAL dressing was anticipated as suitable for PICCs due to its absorbency, nurses experienced difficulty in removing the dressing without dislodging the PICC. This study indicated that its drain dressing design was not conducive to PICCs, which require more frequent dressing changes. Recruited to this arm ceased early due to these concerns and thus further testing was not feasible. We do not recommend this product for PICCs.

Although TA + SPU was viewed positively by clinicians to control haemostasis, repeated TA use at each dressing replacement remained easily removed from skin, but accumulated on the polyurethane catheter, threatening skin injury. Manual removal of TA from PICCs increased the risk of dislodgement, and was time-consuming. While TA has potential benefits at insertion, its use for repeated dressings during PICC dwell was not feasible. Adverse skin complications and phlebitis were most common in the TA group, with pain and tenderness seemingly caused by skin “tugging” with pressure on the PICC. This product requires further testing to determine the effectiveness of applying TA at the initial application only.

The overall failure incidence (9%) was lower than reported in similar adult populations [4, 5], primarily because we censored follow up at a maximum of 4 weeks post insertion. If we had followed patients for the entire PICC dwell (which can be many months), more failures may have been detected. In addition, the enhanced monitoring (e.g. daily inspection) provided by the ReNs (experienced nurses with an expertise in managing vascular access devices) in a trial setting and assistance to clinical staff with changing some of the dressings may have reduced failure incidence.

The most common failure type (6% of all PICCs) was dislodgement, confirming that this is an ongoing, significant problem and that PICC dressing and securement is a priority area for improvement. The multivariable analysis identified two significant factors (male gender, and three or more comorbidities) associated with PICC failure. Male gender may increase risk due to men being more hirsute, which can disrupt dressing adhesiveness [36] and having more muscle movement. In spite of the non-modifiable nature of these two risk factors, clinicians should pay additional attention in ensuring best insertion, monitoring and maintenance practice in men, and those with three or more comorbidities.

Almost one in three patients (29%) had skin complications (rash, blister, itchiness, skin tear, or bruising), and although comparable between treatment arms, this clearly demonstrates the need to improve comfort and skin health in patients with PICCs. Few patients (*n* = 3) developed CABSI and no serious adverse events were caused by the study interventions. Whilst as a pragmatic trial it was not precluded, we did not expect clinical nurses to add additional tapes, dressings, and securements to the extent that was reported (Fig. 1). Bandage, and additional adhesives (typically non-sterile paper tape) were frequent additions in all groups. While reasons for each additional product application were not collected, this might reflect clinicians delaying replacements of the primary dressing (e.g. if a corner was loose) or lacking confidence in its effectiveness. Future pragmatic trials should collect reasons for additional product use and undertake comprehensive cost-benefit analyses of these products.

To our knowledge, this is the first pilot RCT to investigate these three novel securement and dressing interventions in adult patients with PICCs. We acknowledge the design limitations inherent in a pilot RCT with a small sample size. Future RCTs would need 3213 patients/group to test a hypothesis of 10% versus 8% failure, or 1356 patients/group to compare 10% versus 7% failure (stat.ubc.ca/~rollin/stats/ssize/b2.html, 80% power, *p* = 0.05). A comprehensive cost-effectiveness analysis is also needed in future work; in this study we provided initial costs associated with consumables. We did not include thrombosis in the PICC failure endpoint, and although none occurred, we plan to include this in future trials since this is a growing concern in PICCs [8] that may be impacted by poor securement. Despite these limitations, this study demonstrated the safety and feasibility of two regimens and provides preliminary data to inform the design of a larger RCT of CSD + CHG, and TA + SPU (with TA at insertion only) versus standard care (used in this study), to improve PICC outcomes. PICC failure has significant patient and health system costs, it is important that clinicians have evidence available on how to effectively prevent these complications and implement policy decisions about PICC dressing and securement.

## Conclusions

CSD + CHG and TA + SPU (but not PAL + CHG + Tape) are feasible interventions to be tested against controls in a full-scale RCT, to inform measures to prevent PICC failure.

## Abbreviations

BSI: Bloodstream infection
CABSI: Laboratory confirmed bloodstream infection
CHG: Chlorhexidine-gluconate impregnated discs
CRBSI: Catheter-related bloodstream infections
CSD: Combination securement dressing
CVADs: Central venous access devices
HREC: Human Research Ethics Committee
PAL: Polyurethane with absorbent lattice pad
PICCs: Peripherally inserted central catheters
RBWH: Royal Brisbane and Women’s Hospital
RCT: Randomised controlled trial
REDCap: Research Electronic Data CAPture
ReNs: Research Nurses
SPU: Standard polyurethane dressing
SSD: Sutureless securement device
TA: Tissue adhesive
